# Reduced CB1 Cannabinoid Receptor Expression in Alzheimer's Disease and Transgenic Mouse Models

**DOI:** 10.1002/agm2.70080

**Published:** 2026-04-24

**Authors:** Nike von Borcke, Amrei Vivian Purwien, Annik Steiert, Henrike Hasecke, Yvonne Bouter

**Affiliations:** ^1^ Department of Psychiatry and Psychotherapy University Medical Center, Georg‐August‐University Goettingen Germany; ^2^ Department of Nuclear Medicine University Medical Center Göttingen Goettingen Germany

**Keywords:** Alzheimer's disease, Braak stage, endocannabinoid system

## Abstract

**Objectives:**

Therefore, in the present study, the CB1 receptor (CB1R) expression in the hippocampal and cortical tissue of a clinically and neuropathologically characterized cohort of AD patients was analyzed.

**Methods:**

Post‐mortem brain tissue from patients with sporadic AD and non‐demented control subjects was analyzed immunohistochemically, focusing on the hippocampus, medial frontal gyrus, and superior temporal gyrus. CB1R expression levels were measured and correlated with neuropathological hallmarks of AD (amyloid‐β and tau pathology), neuroinflammatory markers (GFAP and IBA1), cognitive status (Reisberg scale), ApoE genotype, and age. Complementary analyzes were performed in two AD mouse models (5xFAD and Tg4‐42).

**Results:**

CB1R expression was significantly reduced in the hippocampus, medial frontal gyrus, and superior temporal gyrus of AD patients. CB1R levels negatively correlated with both amyloid‐β and tau pathology but showed no association with cognitive performance, neuroinflammatory markers, age, or ApoE genotype. Consistent with the human findings, CB1R expression was also reduced in the cortex of 5xFAD mice and in the hippocampus of Tg4‐42 mice.

**Conclusions:**

Our data demonstrate a region‐specific downregulation of CB1R in both human AD brains and transgenic mouse models, which correlates with key neuropathological hallmarks of the disease. These findings suggest a potential role for CB1R in AD pathophysiology and support further investigation into its utility as a biomarker or therapeutic target.

## Graphical abstract

CB1 receptor (CB1R) expression was significantly reduced in the hippocampus, medial frontal gyrus, and superior temporal gyrus of Alzheimer's patients. CB1R levels negatively correlated with amyloid‐β and tau pathology. In addition, CB1R expression was also reduced in the cortex of 5xFAD mice and in the hippocampus of Tg4‐42 mice.

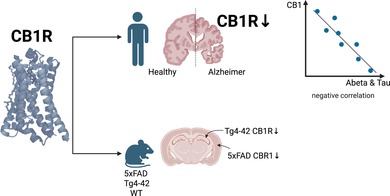

## 1 Introduction

Alzheimer's disease (AD) is a progressive neurodegenerative disorder and the most common cause of dementia, with growing relevance due to an aging global population. Currently, more than 50 million people worldwide are affected by AD, and this number is projected to triple by 2050 [1, 2]. This rising prevalence imposes a substantial socioeconomic burden [3].

Clinically, AD is characterized by a gradual decline in cognitive functions, particularly memory, as well as changes in behavior and personality [4]. The hallmark neuropathological features include the extracellular accumulation of amyloid‐beta (Aβ) plaques, intraneuronal neurofibrillary tangles (NFTs), synaptic dysfunction, and chronic neuroinflammation.

In 1991, Braak and Braak mapped the neuropathological progression of AD by characterizing the spatial distribution of tau and amyloid‐β pathology throughout the brain. They proposed a staging system in which tau pathology is classified into six stages (I–VI), while amyloid‐β deposition is divided into three phases (A–C), each reflecting the hierarchical spread of pathology across distinct brain regions. Specifically, Braak stage 0 corresponds to the absence of neurofibrillary tangles; stages I–II indicate NFT presence in the entorhinal‐perirhinal cortex; stages III–IV mark the extension into limbic structures, including the hippocampus; and stages V–VI indicate widespread NFT distribution throughout the neocortex [5, 6]. Regarding amyloid‐β pathology, stage A is characterized by the initial appearance of extracellular amyloid deposits in the cortical layers of the frontal, temporal, and occipital lobes. As the disease progresses, these deposits extend into most regions of the isocortex (Stage B). By Phase C, amyloid‐β accumulation is widespread and densely aggregated across cortical regions.

AD can be classified into two main forms: sporadic or late‐onset AD (LOAD) and familial AD (FAD). LOAD accounts for approximately 95% of all cases and typically manifests after the age of 60–65 years [7, 8]. In contrast, FAD is much rarer and is characterized by an earlier onset, usually between 30 and 60 years of age [9]. FAD is associated with autosomal dominant mutations in genes encoding the amyloid precursor protein (APP) and presenilin‐1 and ‐2 (PSEN1 and PSEN2) [10]. The major risk factors for LOAD include increasing age, genetic susceptibility, and family history [11, 12]. The ApoE ε4 allele is the strongest known genetic risk factor for sporadic ad [13, 14, 15]. Individuals heterozygous for the ApoE‐ε4 allele have a threefold increased risk of developing AD, while homozygous carriers face an approximately eightfold higher risk [16]. In contrast, carriers of the ApoE ε2 allele are less likely to develop ad [14, 17].

The progression of AD is associated with changes in several biological systems, including the endocannabinoid system (ECS) [18, 19]. The ECS consists of cannabinoid receptors, endogenous ligands (endocannabinoids), and the enzymes involved in their synthesis and degradation [20]. The ECS plays a crucial role in numerous physiological processes, including cognition, emotional regulation, appetite, pain perception, and neuronal plasticity [21, 22]. The most well‐characterized endocannabinoids are N‐arachidonoylethanolamine (AEA) and 2‐arachidonoylglycerol (2‐AG), both of which act as lipid signaling molecules [23, 24].

The two main cannabinoid receptors, CB1 and CB2, are G‐protein‐coupled receptors with distinct expression patterns. CB1 receptors (CB1R) are predominantly located in the central nervous system, particularly in the basal ganglia, hippocampus, frontal cortex, and mesolimbic dopamine (DA) system [25, 26, 27]. In contrast, CB2 receptors are predominantly present in the immune system and peripheral tissues, where they play a role in modulating inflammatory responses [28, 29]. CB2 receptors are also expressed in the central nervous system, though to a lesser extent, and are mainly localized to microglia and other immune‐related cells within the brain [30, 31].

Studies suggest that several components of the ECS are altered in individuals with AD, including the enzymes responsible for endocannabinoid metabolism. For example, anandamide (AEA) levels are reduced in the cortex of AD patients, showing an inverse correlation with Aβ42 concentrations and cognitive impairment [32]. In contrast, in the hippocampus, the activity of enzymes that degrade 2‐arachidonoylglycerol (2‐AG) is elevated [33], potentially leading to dysregulated 2‐AG levels and altered CB1 receptor signaling. Furthermore, the ECS is discussed as a promising pharmacotherapeutic target in AD due to its role in modulating neuroinflammation and cognitive function [28, 34, 35].

Despite growing interest in the role of the endocannabinoid system in AD, the status of CB1 receptors in the AD brain remains inconclusive. Some studies have reported reduced CB1 receptor immunoreactivity and ligand binding, while mRNA expression levels appear unchanged [36, 37]. In contrast, other findings suggest no significant alterations in CB1 receptor expression [38], or increased CB1 receptor protein expression [39]. Moreover, the relationship between CB1 receptor alterations and cognitive decline in AD has yet to be clearly defined. In the present study, we examined CB1 receptor expression in the hippocampal and cortical tissue of a clinically and neuropathologically characterized cohort of AD patients.

## 2 Materials and Methods

### 2.1 Human Samples

Brain tissue samples from the superior temporal gyrus, medial frontal gyrus, and hippocampus were obtained from patients with sporadic AD and non‐demented control subjects via the Netherlands Brain Bank (NBB, Amsterdam, The Netherlands) [40]. Baseline characteristics are shown in Table 1. The NBB performs autopsies within 4–10 h post‐mortem, thereby minimizing tissue degradation. Written informed consent for brain donation and use of medical data for research purposes was obtained from all donors. Alzheimer's disease diagnoses were confirmed post‐mortem based on established clinical and neuropathological criteria. Control subjects had no history of psychiatric or neurological disorders, brain metastases, or prior psychotropic or neuroactive medication. Their classification as controls was based on a comprehensive review of medical records and confirmation of Braak stage (0–3) by the NBB. Cognitive status of all sporadic AD cases was evaluated using the Reisberg Global Deterioration Scale (GDS). The Reisberg scale is a clinical tool used to assess the severity of cognitive decline and is divided into seven stages. Stages 1–3 represent the pre‐dementia phases, while stages 4–7 correspond to progressive stages of dementia [41]. For each donor, the GDS score was assigned by the treating physician shortly before death, reflecting the individual's cognitive function at the end of life. All experiments involving human tissue were approved by the Ethics Committee of the University Medical Center Göttingen (protocol number 12/1/15).

**TABLE 1:** Patient characteristics.

	Characteristics	AD	Control
Superior temporal gyrus	Samples	*n* = 41	*n* = 15
	Sex (female/male)	36/5	8/7
	Age (± SEM)	79.85 ± 2.073	82.8 ± 2
	Braak stage	4–6	0–3
	ApoE4 carriers [homozygous]	21 [5]	3 [0]
	Reisberg Global Deterioration Scale	2–7	n.a.
	Post‐mortem delay (min)	336.3 ± 21.7	366.5 ± 27.16
Medial frontal gyrus	Samples	*n* = 23	*n* = 10
	Sex (female/male)	16/7	4/6
	Age (± SEM)	85.35 ± 1.604	80.40 ± 2.495
	Braak stage	4–6	0–1
	ApoE4 carriers [homozygous]	12 [1]	2 [0]
	Reisberg Global Deterioration Scale	2–7	n.a.
	Post‐mortem delay (min)	344.2 ± 31	493.9 ± 88.4
Hippocampus	Samples	*n* = 20	*n* = 10
	Sex (female/male)	15/5	4/6
	Age (± SEM)	87.80 ± 0.9050	79.85 ± 2.073
	Braak stage	4–6	0–1
	ApoE4 carriers [homozygous]	10[1]	2 [0]
	Reisberg Global Deterioration Scale	2–7	n.a.
	Post‐mortem delay (min)	334.8 ± 35.8	410.1 ± 32.65

### 2.2 Mouse Models

In this study, two established AD‐like transgenic mouse lines were used: 5xFAD [42], and Tg4‐42 [43]. 5xFAD mice overexpress the 695‐amino‐acid isoform of human amyloid precursor protein (APP695) harboring the Swedish (K670N/M671L), London (V717I), and Florida (I716V) mutations, under the control of the murine Thy1 promoter. Additionally, they express human presenilin‐1 (PSEN1) with the M146L and L286V mutations, also driven by the Thy1 promoter. Female mice aged five and 9 months and age‐matched wild‐type controls were included in the study. Tg4‐42 mice express human Aβ4–42 fused to the murine thyrotropin‐releasing hormone (TRH) signal peptide under the control of the neuron‐specific Thy1 promoter. In this study, female Tg4‐42 mice aged 8 months were included, alongside age‐matched wild‐type controls. All mice used in the study had a C57BL/6J genetic background (Jackson Laboratory, Bar Harbor, ME, USA). Mice were sacrificed through transcardial perfusion. Brain samples were carefully dissected and post‐fixed in 4% phosphate‐buffered formalin at 4°C. All animal procedures complied with German animal welfare regulations and were approved by the local authorities (LAVES) [15/1760, 16/2364, 14/1450].

### 2.3 Immunohistochemistry

The paraffin‐embedded tissue was cut into 5 μm sections. Immunohistochemical staining was performed as previously described [44]. In brief, 3,3′‐diaminobenzidine (DAB) staining was carried out with the antibody CB1 (1:200; Abcam, Cambridge, UK). A corresponding secondary biotinylated anti‐rabbit antibody was used (1:200; Jackson ImmunoResearch Laboratories Inc., West Grove, USA). Visualization was achieved using the Vectastain ABC Kit (Vector Laboratories, Burlingame, CA, USA), with DAB serving as the chromogen.

For fluorescence immunostaining, the primary antibodies GFAP (1:500; Synaptic Systems, Göttingen, Germany) and IBA1 (1:300; Synaptic Systems, Göttingen, Germany) were used to analyse reactive astrocytes and microglia, respectively. NeuN (1:500; Synaptic Systems, Göttingen, Germany) was used to identify neuronal nuclei. As secondary antibodies, anti‐rabbit Alexa FluorPlus 488 (1:200; Thermo Fisher Scientific Inc., Carlsbad, CA, USA) and anti‐guinea pig Alexa Fluor 594 (1:200; Thermo Fisher Scientific Inc., Carlsbad, CA, USA) were applied.

An Olympus BX51 microscope equipped with a Moticam Pro 282B digital camera was used to capture images of CB1‐stained sections. Quantitative analysis was performed using ImageJ software (version 1.53k; Rasband, U.S. National Institutes of Health, Bethesda, MD, USA). Three representative pictures were captured from the superior temporal gyrus (20×), medial frontal gyrus (20×), and hippocampus (4×), respectively. A fixed threshold was applied to the DAB‐stained sections, and the CB1‐positive area was calculated relative to the total image area.

For fluorescence image quantification, brain scans were acquired using a Nikon Eclipse Ti confocal microscope in combination with NIS‐Elements AR software at 40× magnification. Imaging parameters (exposure time, brightness, and gain) were kept constant across all samples. For image analysis, ImageJ was used. Scanned images of inflammation markers were separated into individual fluorescence channels, converted to 8‐bit grayscale, inverted, and thresholded to quantify the positive staining signal.

The proportion of stained area relative to the total analyzed image area was calculated. The number of immunopositive cells for NeuN was calculated using an average of three fields of view per area.

To ensure unbiased analysis, all quantification procedures were performed with the evaluator blinded to the experimental conditions.

### 2.4 Statistical Analysis

Group differences were analyzed using the Mann–Whitney *U* test or unpaired *t*‐test. Spearman correlation was used to assess associations between variables. The following significance levels were determined: ***p < 0.001, **p < 0.01, *p < 0.05. GraphPad Prism 5 (GraphPad Software, San Diego, CA, USA) was applied for the statistical analysis.

## 3 Results

### 3.1 Reduced CB1R Expression in the Cortex and Hippocampus of AD Patients

**FIGURE 1:**
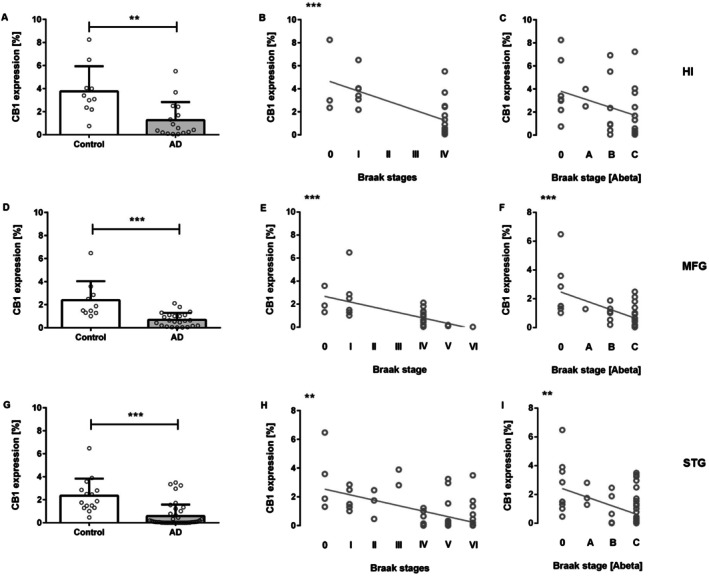
Reduced CB1R Expression in AD patients. CB1R expression was significantly reduced in the (A) hippocampus, (D) medial frontal gyrus, and (G) superior temporal gyrus of AD patients. In the hippocampus, CB1R levels negatively correlated with (B) Braak stage (tau pathology), but not with (C) Aβ pathology. In the medial frontal gyrus and superior temporal gyrus, CB1R expression showed significant negative correlations with Braak stages representing both (E, F) tau and (H, I) Aβ pathology. (A, D, G): Mann–Whitney test; Data presented as mean ± SEM; (B, C, E, F, H, I): Spearman correlation. **p < 0.01, ***p < 0.001. HI, hippocampus; MFG, medial frontal gyrus; STG, superior temporal gyrus.

DAB staining using a primary antibody against CB1 revealed a significant reduction in CB1 receptor expression in the superior temporal gyrus (STG), medial frontal gyrus (MFG), and hippocampus (HI) of AD patients (Figure 1A,D,G: Mann–Whitney test: STG: *p* < 0.001; MFG: *p* < 0.001; HI: *p* = 0.002). Figure 6 presents representative immunohistochemical images illustrating CB1R expression in AD patients and control.

### 3.2 CB1R Expression Correlates With AD Pathology

Correlation analyses were performed to examine the relationship between CB1 receptor expression and AD pathology, specifically tau and amyloid‐β accumulation, as assessed by Braak staging. CB1R expression was negatively correlated with tau pathology, showing that lower CB1R levels were associated with higher tau accumulation in all three analyzed brain regions (Figure 1B,E,H: Spearman correlation: STG: *r* = −0.5337, *p* < 0.01; MFG: *r* = −0.623, *p* < 0.001; HI: *r* = −0,635, *p* < 0.001). Similarly, CB1R expression negatively correlated with Aβ pathology in both the *superior temporal gyrus and medial frontal gyrus* regions (Figure 1C,F,I: Spearman correlation: STG: *r* = −0.487, *p* < 0.01; MFG: *r* = −0.570, *p* < 0.001; HI: *r* = −0.355, *p* = 0.053). These findings suggest that CB1R expression declines with increasing pathological burden in AD.

### 3.3 No Correlation Between CB1R Expression and Markers of Neuroinflammation

**FIGURE 2:**
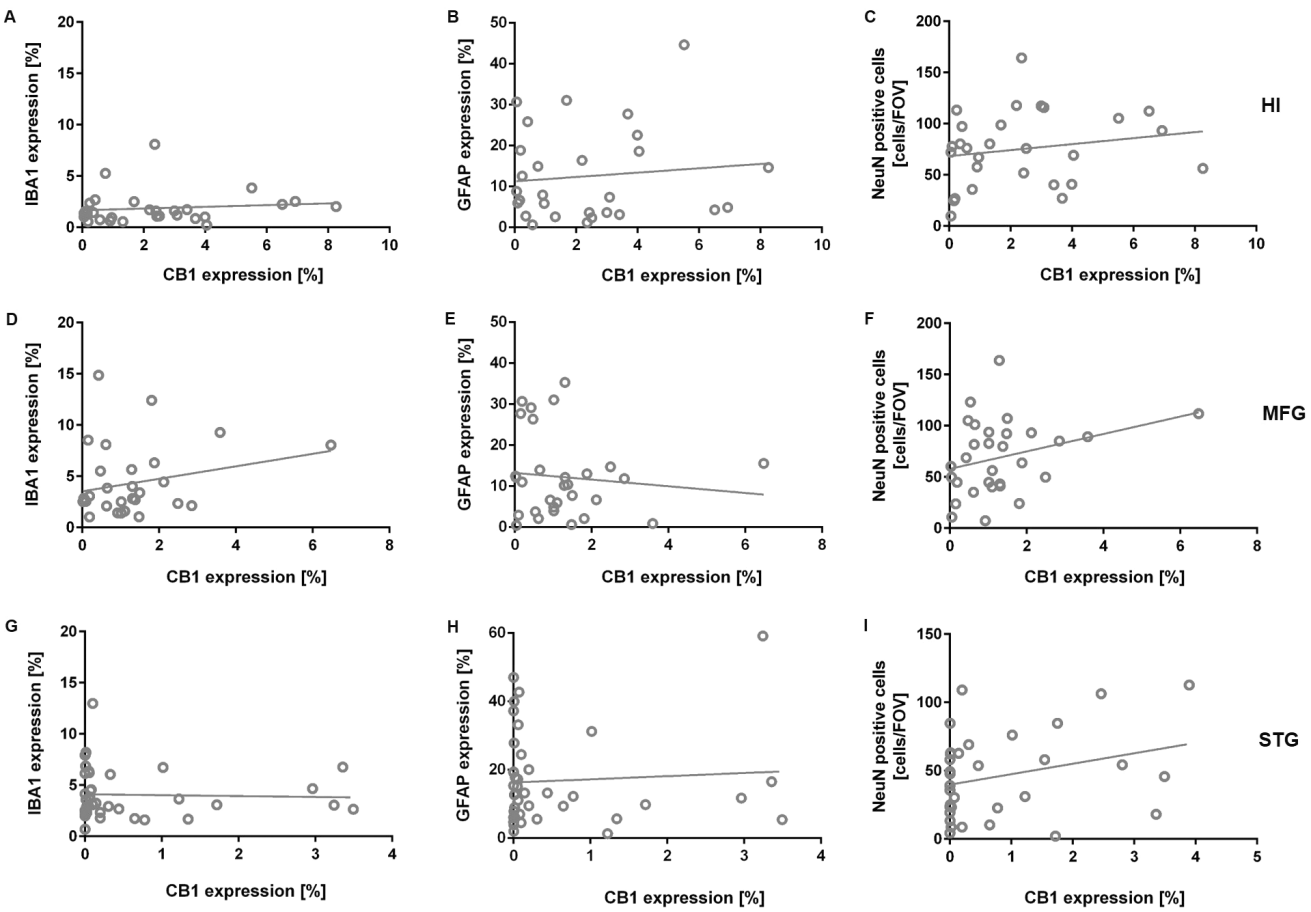
No Correlation between CB1R Expression and Neuroinflammation Markers or Neuron number. CB1R expression did not correlate with GFAP (a marker of reactive astrocytes) or IBA1 (a marker of microglia) in the (A, B) hippocampus, (D, E) medial frontal gyrus, or (G, H) superior temporal gyrus. Furthermore, neuronal cell number did not correlate with CB1R expression in the hippocampus (C), medial frontal gyrus (F), or superior temporal gyrus (I). Spearman correlation. FOV, field of view; HI, hippocampus; MFG, medial frontal gyrus; STG, superior temporal gyrus.

To assess neuroinflammation, we quantified glial fibrillary acidic protein (GFAP) as a marker of reactive astrocytes and ionized calcium‐binding adapter molecule 1 (IBA1) as a marker of microglia. CB1 receptor immunoreactivity in both the cortex and hippocampus showed no significant correlation with GFAP or IBA1 expression (Figure 2: Spearman correlation: GFAP: STG: *r* = −0.090, *p* = 0.582; MFG: *r* = −0.069, *p* = 0.714; HI: *r* = −0.013, *p* = 0.947; IBA1: STG: *r* = −0.032, *p* = 0.843; MFG: *r* = −0.123, *p* = 0.508; HI: *r* = 0.164, *p* = 0.393).

### 3.4 No Correlation Between CB1R Expression and Neuron Numbers

AD samples showed a reduced number of neurons, as indicated by NeuN immunostaining, a neuronal‐specific nuclear marker (Figure S1: Mann–Whitney test: STG: *p* < 0.001; MFG: *p* = 0.045; HI: *p* = 0.134). However, the neuronal cell number did not correlate with CB1R expression in the hippocampus, superior temporal gyrus, and medial frontal gyrus (Figure 2C,F,I: Spearman correlation: NeuN: STG: *r* = 0.290, *p* = 0.100; MFG: *r* = 0.306, *p* = 0.099; HI: *r* = 0.185, *p* = 0.343).

### 3.5 No Correlation Between CB1R Expression and Demographic or Disease‐Related Variables

**FIGURE 3:**
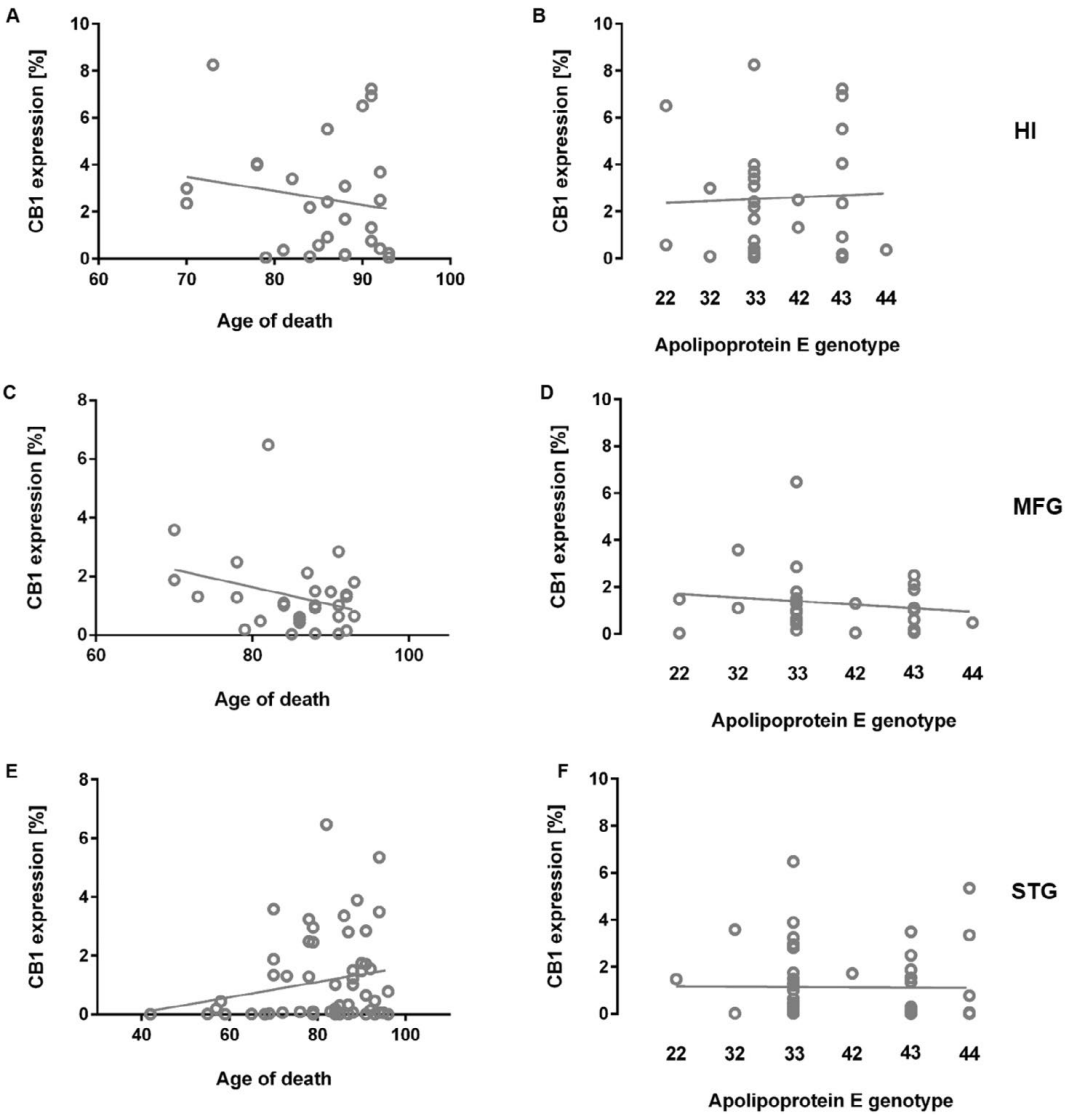
No Correlation of CB1R Expression with Age and ApoE Genotype. CB1R expression did not correlate with age in the (A) hippocampus, (C) medial frontal gyrus, or (E) superior temporal gyrus. Similarly, no correlation was observed between CB1R expression and (B, D, F) ApoE genotype in any of the examined brain regions. Spearman correlation. HI, hippocampus; MFG, medial frontal gyrus; STG, superior temporal gyrus.

CB1 receptor immunoreactivity in the cortex and hippocampus did not correlate with age at death (Figure 3A,C,E: Spearman correlation: STG: *r* = 0.206, *p* = 0.123; MFG: *r* = −0.180, *p* = 0.341; HI: *r* = −0.150, *p* = 0.429). Furthermore, no significant association was observed between CB1R expression and ApoE genotype (Figure 3B,D,F: Spearman correlation: STG: *r* = −0.046, *p* = 0.731; MFG: *r* = −0.150, *p* = 0.426; HI: *r* = 0.054, *p* = 0.774). Moreover, there were no sex‐related differences in CB1R expression in either brain region (Mann–Whitney *U* test: STG: *p* = 0.448; MFG: *p* = 0.702; HI: *p* = 0.734).

### 3.6 No Correlation of Post‐Mortem CB1R Expression With Pre‐Death Cognitive Status

**FIGURE 4:**
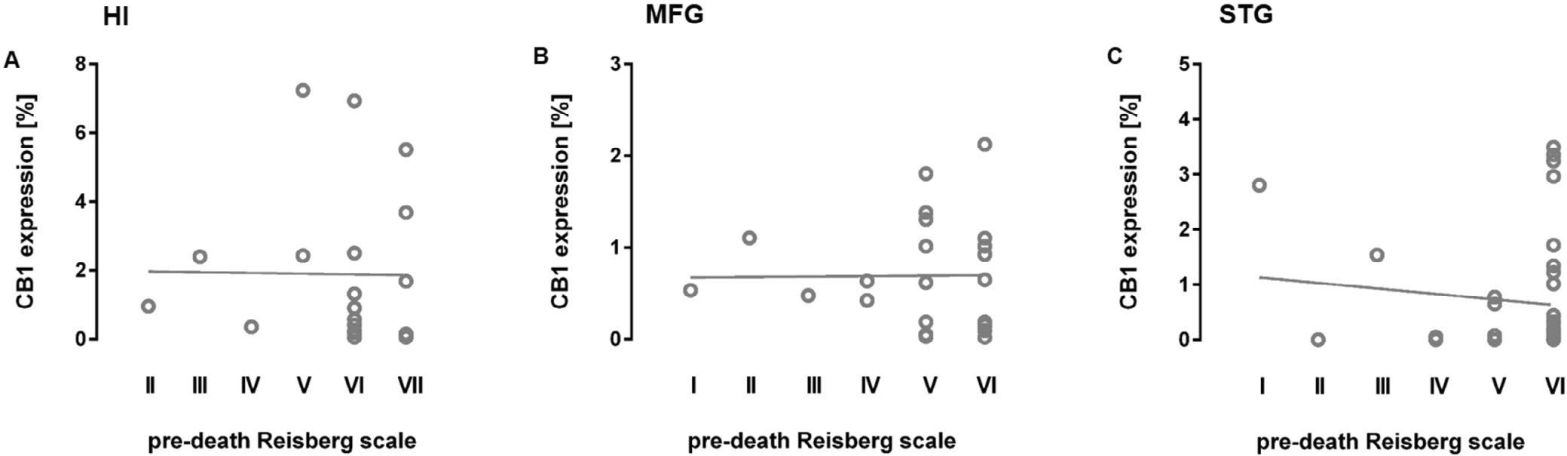
No Correlation of CB1R Expression and Cognitive Status in AD patients. CB1R expression did not correlate with pre‐death cognitive status, as assessed by the Reisberg Scale, in the (A) hippocampus, (B) medial frontal gyrus, or (C) superior temporal gyrus of AD patients. Spearman correlation. HI, hippocampus; MFG, medial frontal gyrus; STG, superior temporal gyrus.

Correlation analyses were performed to investigate the relationship between post‐mortem CB1 receptor immunoreactivity and cognitive performance prior to death, as measured by the Reisberg scale. No significant correlation was found between CB1R expression in the analyzed brain regions and cognitive scores (Figure 4: Spearman correlation: STG: *r* = 0.106, *p* = 0.523; MFG: *r* = −0.004, *p* = 0.848; HI: *r* = −0.208, *p* = 0.378).

### 3.7 Reduced CB1R Expression in AD Mouse Models

**FIGURE 5:**
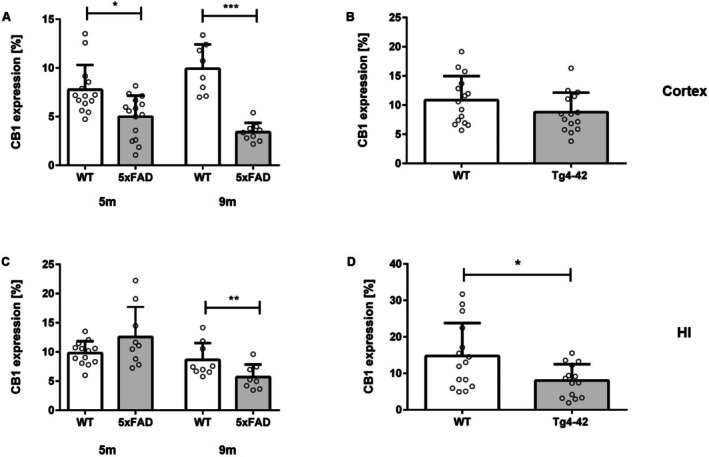
Reduced CB1R Expression in AD Mouse Models. CB1R expression was reduced in the (A) cortex of 5‐ and 9‐month‐old 5xFAD mice and in the (C) hippocampus of 9‐month‐old 5xFAD mice. In Tg4‐42 mice, CB1R expression was significantly decreased in the (D) hippocampus, while expression in the (B) cortex remained unchanged (unpaired *t*‐test: Cortex: *p* = 0.1427; hippocampus: *p* = 0.0179). Unpaired *t*‐test, *n* = 8–13 per group. Data presented as mean ± SEM; *p < 0.05, **p < 0.01. HI, hippocampus.

**FIGURE 6:**
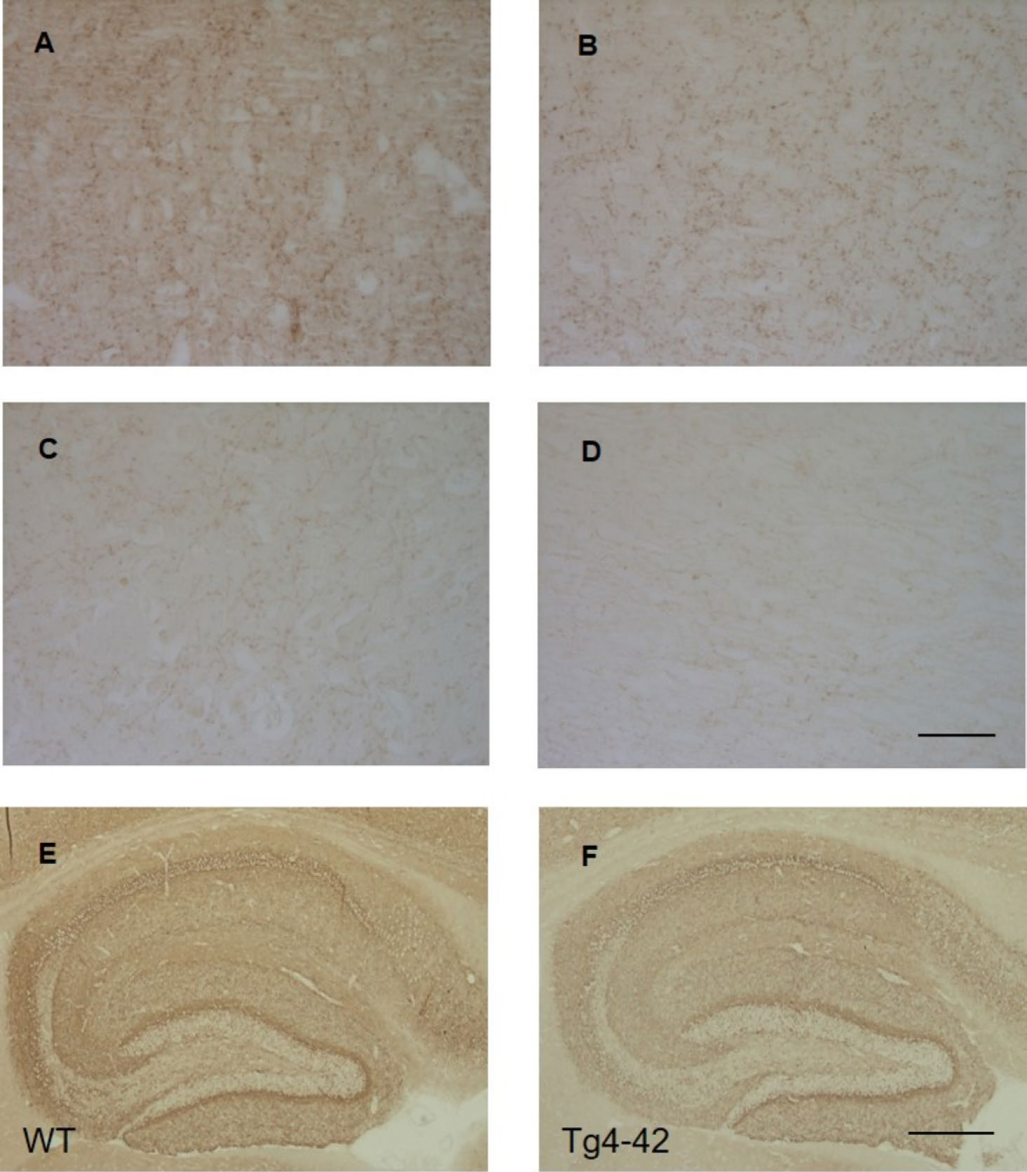
CB1R expression in human AD and mouse brain tissue. Representative images showing reduced CB1R immunoreactivity in cortical regions of AD patients (B, C, D) compared to a control (A). Reduced CB1R in the hippocampus of Tg4‐42 mice compared to a WT control. Scale bar = 100 μm.

CB1R expression was assessed in 5xFAD and Tg4‐42 transgenic AD mice. In 5xFAD mice, characterized by extensive extracellular Aβ deposition, CB1R expression was significantly reduced in the cortex but remained unchanged in the hippocampus at 5 months of age (Figure 5; unpaired *t*‐test: cortex, *p* = 0.003; hippocampus, *p* = 0.094). By 9 months, an age‐dependent decline in hippocampal CB1R expression became evident (unpaired *t*‐test: hippocampus, *p* = 0.003). In contrast, Tg4‐42 mice, which predominantly exhibit strong intraneuronal Aβ accumulation in the hippocampus, showed a reduction in CB1R expression limited to the hippocampal region (Figures 5, 6E,F: unpaired *t*‐test: *cortex*: *p* = 0.142; *hippocampus*: *p* = 0.017).

## 4 Discussion

A growing body of evidence suggests that disruptions in the endocannabinoid system (ECS) contribute to the pathophysiology of AD, and that ECS‐targeted therapies may hold disease‐modifying potential [45, 46]. However, findings regarding CB1 receptor (CB1R) expression in AD are inconsistent (Table 2). Therefore, the aim of this study was to investigate CB1R expression in the brains of AD patients in relation to tau and Aβ deposition, cognitive function, inflammation, and ApoE genotype.

**TABLE 2:** CB1 receptor expression changes in Alzheimer's disease patients.

Group characteristics (number of cases; sex; AD stage)	Brain area	Technique	Results	References
*n* = 16 (AD: 11, C: 5) Gender: N/A Three groups: ○ Braak stage I–II ○ Braak stage III–IV ○ Braak stage V–VI	Prefrontal cortex	Autoradiography (with [^125^I]SD‐7015)	Increased CB1 receptor protein expression: ○ CB1 expression: controls<AD Braak stage V–VI <AD Braak stage III–IV <AD Braak stage I–II	[39]
*n* = 53 (AD: 36; C: 17) Gender: N/A Braak stage I–VI	Frontal cortex Amygdala Basal forebrain Striatum Hippocampus Enthorinal cortex	Autoradiography (with [35S]GTPɤS and WIN55,212‐2)	Altered CB1 receptor protein expression: ○ Braak I–II: increased expression in areas of hippocampus and frontal cortex (layer VI) ○ Braak III–IV: increased expression in areas of hippocampus and frontal cortex (layer VI) ○ Braak V–VI: decreased expression in hippocampus	[47]
*n* = 8 (AD: 5; C: 3) Gender: ○ AD: 3♂ 2♀ ○ Control: 2♂ 1♀ AD patients met DSM‐III‐R criteria for dementia	Hippocampus Neocortex Basal ganglia Parts of the brainstem	Autoradiography (with [3H]CP‐55,940)	Reduced CB1 receptor protein expression in hippocampus, caudate, substantia nigra and internal segment of globus pallidus No alterations in other neocortical and basal ganglia structures	[36]
*n* = 11 (AD: 6; C: 5) Gender: ○ AD: 3♂ 3♀ ○ Control: 3♂ 2♀ Clinically and pathologically confirmed AD patients	Frontal cortex	[35S]GTPɤS binding assay, Western Blot	Reduced CB1 receptor protein expression	[37]
*n* = 31 (AD: 15; C: 16) Gender: ○ AD: 5♂ 10♀ ○ C: 7♂ 9♀ Braak stages V/VI Cognitive status assessed (MMSE)	Frontal cortex	Western Blot	Reduced CB1 receptor protein expression No correlation of CB1 with AD markers (Aβ_42_ or senile plaques) or cognitive status	[48]
*n* = 33 (AD: 17, C = 16) Gender: ○ AD: 9♂ 8♀ ○ C: 11♂ 5♀ Braak stages III–VI Cognitive status assessed of majority (MMSE, CAMCOG)	Cortex Hippocampus Anterior cingulate Caudate	Autoradiography (with [(3)H]SR141716A), Western Blot	Unaltered CB1 receptor protein expression Frontal CB1 levels correlated with pre‐death MMSE and CAMCOG in AD	[49]
*n* = 28 (AD: 18; C: 10) Gender: N/A Braak stages III/IV and VI	Hippocampus	Western Blot	Unaltered CB1 receptor protein expression	[33]
*n* = 18 (AD: 11, C: 7) Gender: ○ AD: 3♂ 8♀ ○ C: 3♂ 4♀ MMSE assessed: mild to moderate cases (11–24)	Cortex Insula Cingulum Striatum	PET in vivo (with [^18^F]MK‐9470 PET and [^11^C]PIB PET)	Unaltered CB1 receptor protein expression No correlation of CB1 with MMSE	[50]

Abbreviations: AD, Alzheimer's Disease; C, Control; CAMCOG, Cambride Cognitive Examination; DSM‐III‐R, Diagnostic and Statistical Manual of Mental Disorders; MMSE, Mini Mental State Examination; PET, Positron emission tomography.

We observed a reduction in CB1 receptor expression in both the cortex and hippocampus of AD patients. These findings align with previous post‐mortem studies reporting decreased CB1R expression, particularly in advanced stages of the disease across various brain regions [36, 37, 47, 48]. For example, Westlake et al. reported reduced CB1R protein levels in the hippocampus, substantia nigra, and internal segments of the globus pallidus, whereas no significant changes were detected in the neocortex or basal ganglia [36]. Similarly, Ramirez et al. found a decline in CB1R expression in the frontal cortex of AD patients [37]. Notably, Manuel et al. reported that CB1R expression varies depending on the degree of neuropathological burden. Specifically, they found increased expression in Braak stages I–II, while a significant reduction was observed in later stages (V–VI) compared to both healthy controls and intermediate stages (III–IV) [47]. This is in line with our findings, as we could demonstrate a negative correlation between Braak stage and CB1 expression, suggesting that CB1R alterations are closely associated with the progression of AD pathology.

Interestingly, several studies have found no significant differences in CB1 receptor expression between individuals with AD and healthy controls, using a variety of methodological approaches such as PET imaging, immunoblotting, autoradiography, Western blotting, and immunofluorescence [33, 49, 50]. Among these, only Mulder et al. accounted for disease progression by stratifying cases based on Braak staging. Notably, the PET study by Ahmed et al. involved AD patients with relatively mild clinical symptoms, as indicated by higher cognitive scores. This may partly explain the absence of significant CB1R alterations, considering the potential for receptor upregulation during early stages of the disease.

Two previous studies have reported an upregulation of CB1R expression during the early stages of AD (Braak stages I–II) [39, 47]. The authors proposed that this increase may represent a compensatory mechanism in response to early pathological changes. This hypothesis is supported by findings from Aso et al., who observed elevated CB1R expression in the neocortex of 3‐month‐old APP/PS1 mice, followed by a progressive, age‐dependent decline starting at 6 months, coinciding with the accumulation of AD‐related pathology [51]. Interestingly, wild‐type mice also exhibited an age‐related reduction in CB1R expression by 12 months of age, suggesting that both disease progression and aging may influence CB1R dynamics.

Taken together, these findings—including our own—suggest that CB1 receptor signaling may be impaired in advanced stages of AD pathology, potentially contributing to the progression of neurodegeneration.

While we observed a correlation between CB1 receptor expression and Braak staging—reflecting Tau and Aβ pathology—we did not find a significant association between CB1R levels and markers of neuroinflammation, which represents another core feature of AD. This finding is not unexpected, given the cellular distribution of cannabinoid receptors. Unlike CB2 receptors, which are primarily expressed on microglia and play a well‐established role in modulating neuroinflammatory responses, CB1 receptors are predominantly localized on neurons, with only moderate expression on astrocytes and microglia [52, 53, 54, 55]. Although some studies have suggested a potential role for CB1R expression in immune signaling within the brain, its direct involvement in regulating microglial activity and neuroinflammation remains less well‐characterized [56, 57]. Accordingly, the absence of a correlation between CB1R and inflammatory markers in our dataset may reflect these underlying differences in receptor function and cellular localization.

The ECS plays a crucial role in cognitive functions [58]. Previous studies have suggested a potential association between CB1 receptor availability and cognitive performance in AD. In preclinical models, deletion of CB1R in APP/PS1 mice resulted in an accelerated onset of memory impairments, indicating a protective role of CB1R in cognitive decline [59]. Furthermore, studies in WT mice have shown that the age‐related decline in cognitive function is accelerated in the absence of CB1 receptors [60]. Clinically, Lee et al. reported no overall differences in CB1R expression between AD patients and healthy controls; however, within the AD group, individuals with higher CB1R levels in the frontal cortex showed better cognitive performance. The authors hypothesized that this could be due to enhanced modulation of glutamate release and better control of excitotoxicity [49]. In contrast, our study did not find a significant association between CB1R expression and cognitive performance, as measured by pre‐mortem Reisberg scores. These findings align with those of Ahmad et al., who also observed no correlation between CB1R availability and cognitive function using PET imaging. However, it should be noted that the use of global cognitive measures such as the Reisberg score may lack the sensitivity to detect subtle region‐specific associations between CB1R expression and distinct cognitive domains.

We also investigated the relationship between CB1 receptor expression and ApoE genotype. ApoE, a key regulator of lipid metabolism, represents the most prominent genetic risk factor for late‐onset AD. Carriers of the ApoE‐ε4 allele have a higher risk of developing AD than individuals with the more prevalent ApoE‐ε3 variant [61, 62]. Emerging evidence suggests that ApoE genotype also influences endocannabinoid signaling [63, 64]. Specifically, ApoE‐ε4 carriers exhibit distinct alterations in endocannabinoid signaling and lipid profiles. It has been shown that both human and murine AD brains expressing the ApoE‐ε4 allele show decreased levels of synaptamide and anandamide, alongside elevated concentrations of 2‐arachidonoylglycerol (2‐AG), when compared to ApoE‐ε3 carriers [63]. These findings highlight a genotype‐dependent dysregulation of the endocannabinoid system in the context of AD. However, to date, evidence linking the ApoE ε4 allele to alterations in CB1R expression remains limited. In our study, we observed no significant association between CB1R expression and ApoE ε4 genotype. These findings are consistent with those of Ahmad et al., who also reported no correlation between CB1R availability and ApoE genotype using the CB1‐specific PET tracer [18F]MK‐9470 [50].

Alterations in the ECS have been reported in various AD mouse models, and consistent with our findings, reduced CB1 receptor expression has been described across several of these models (see Table 3). Similarly, in line with our observations in human brain tissue, we detected decreased CB1 receptor expression in both female 5xFAD and Tg4‐42 transgenic mice. Specifically, CB1R expression was significantly decreased in the cortex of 5‐ and 9‐month‐old 5xFAD mice, as well as in the hippocampus of 9‐month‐old 5xFAD mice. Similarly, Medina‐Vera et al. reported reduced hippocampal CB1R expression in 11‐month‐old 5xFAD mice. They also detected elevated levels of diacylglycerol lipase α (DAGLα), the enzyme responsible for synthesizing the endocannabinoid 2‐arachidonoylglycerol (2‐AG) [68]. Notably, Medina‐Vera et al. examined a mixed group of male and female mice, whereas our analysis was restricted to females.

**TABLE 3:** CB1 receptor expression changes in animal models of Alzheimer's disease.

Mouse model	Age	Brain area	Technique	Results	References
Male 3xTg	2, 6, 12 months	Prefrontal cortex Prelimbic cortex Dorsal hippocampus Ventral hippocampus Basolateral amygdala complex	In situ hybridization & immunohistochemistry	2 months: no changes 6 and 12 months: significantly higher CB1 mRNA levels in prefrontal cortex, dorsal hippocampus, and basolateral amygdala complex 12 months: CB1 receptor immunoreactivity decreased in basolateral amygdala and dorsal hippocampus	[65]
Male 3xTg	6 months	Multiple brain regions	Autoradiography (with WIN55‐mediated 35S‐GTPγS binding), immunohistochemistry	CB1 ↓ in amygdala and VI layer of motor cortex	[66]
Hippocampal astrocytes from 3xTg	2–3 days	Primary cultures of astrocytes from the hippocampus of 3xTg	HPLC	Female specific decrease in CB1 gene expression	[67]
Male & female heterozygous & homozygous 5xFAD	11 months	Hippocampus	Western Blot	Reduction of CB1 receptor protein expression	[68]
Male APPswe/PS1dE9	10–12 months	Hippocampus	Immunohistochemistry	Significant reduction in the number of CB1‐IR hippocampal neurons	[69]
Male APPswe/PS1dE9	3, 6, 12 months	Neocortex	Immunohistochemistry and quantitative densitometry	3 months: CB1 ↑ 6 months: CB1 ↓ 12 m:CB1 ↓	[51]
Female APPswe/PS1dE9	13–14 months	Hippocampus Entorhinal cortex Medial frontal cortex	Functional autoradiography	Unaltered cannabinoid CB1 receptor signaling	[70]
Male and female APPswe/PS1dE9	3 months	Prefrontal cortex Hippocampus Cerebellum Olfactory bulb Hypothalamus	Quantitative Real‐Time PCR	Male: altered CB1R mRNA levels ○ Hippocampus, prefrontal cortex ↓ ○ Hypothalamus ↑ Female: altered CB1R mRNA levels ○ Olfactory bulb, prefrontal cortex, hypothalamus ↑	[71]
Male and female APP/PS1‐21	6, 9, 12, 15 months	Whole brain	PET imaging, autoradiography, immunohistochemistry, Western Blot	Age‐dependent lower binding ratios of the inverse agonist for CB1R, [18F]FMPEP‐d2, in the parietotemporal cortex and hippocampus (PET, autoradiography)	[72]
Tg2576	4 months	Hippocampus	Western Blot	Unaltered CB1 expression, however altered membrane localization and function	[73]

Abbreviations: HPLC, High Performance Liquid Chromatography; IR, immunoreactivity; m, months; PET, positron emission tomography.

Sex differences in both AD pathology and the ECS are well documented. Studies indicate that females—both in humans and rodent models—generally exhibit more pronounced AD‐related neuropathology [74, 75, 76]. Additionally, CB1 receptor availability has been reported to be lower in women [77]. For instance, female 5xFAD mice consistently develop earlier and more severe pathology compared to males [75, 78, 79]. These robust sex differences highlight the importance of not mixing sexes within experimental groups. Nevertheless, we recognize the value of investigating sex as a biological variable, and future studies should include male animals to explore potential sex‐specific differences in CB1R expression.

Moreover, we observed reduced CB1 expression in the hippocampus of 8‐month‐old Tg4‐42 mice. These animals exhibit strong intraneuronal Aβ accumulation, particularly within the CA1 pyramidal layer, with only minimal Abeta pathology in the cortex [80]. Our findings suggest that Aβ accumulation may contribute to region‐specific alterations in CB1 receptor expression. Supporting this, Bedse et al. reported age‐ and region‐specific alterations of CB1 expression as well as an inverse correlation between Aβ levels and CB1 receptor protein expression in male 3xTg mice [65]. The decline in CB1 receptor expression in the hippocampus of Tg4‐42 mice may contribute to the pronounced hippocampus‐dependent memory deficits reported in this model [81, 82, 83]. Notably, female Tg4‐42 mice display severe behavioral and memory impairments by 8 months of age, coinciding with the observed decline in hippocampal CB1R expression. It has been shown that CB1 receptors play a critical role in learning and memory processes [84, 85, 86]. Next to AD deregulation of the ECS has been observed in a range of neurological disorders associated with cognitive impairments, including Parkinson's disease [87], Huntington's disease [88, 89, 90], and amyotrophic lateral sclerosis [91].

Furthermore, genetic deletion of CB1 receptors in different transgenic mouse models of AD has been associated with aggravated cognitive deficits [59, 92], supporting the hypothesis that CB1 receptor signaling may exert a neuroprotective role in AD pathology. These findings highlight the therapeutic potential of modulating CB1 receptors with exogenous cannabinoids as a strategy to slow disease progression and preserve cognitive function. We have previously demonstrated that therapeutic treatment with the synthetic cannabinoid WIN 55,212–2 rescues memory deficits and reduces Aβ plaque pathology in 5XFAD mice [93], and also improves cognition and locomotor activity in Tg4‐42 mice [34]. Furthermore, chronic treatment with the CB1 receptor agonist arachidonyl‐2‐chloroethylamide (ACEA) has been shown to alleviate cognitive impairments and exert neuroprotective effects in AβPP/PS1 mice [51].

A major limitation of our study is its post‐mortem design, which entails potential issues such as protein degradation, a limited selection of brain regions for analysis, and difficulties in linking molecular data with cognitive assessments obtained during the patients' lifetimes. An additional limitation is that IBA1 and GFAP signals were not normalized to Aβ or tau aggregates. Since gliosis often occurs in close association with pathological deposits, our measurements reflect overall regional glial reactivity rather than plaque‐ or tangle‐specific responses. Future studies using co‐localization or normalization approaches will be needed to address this limitation more directly. A further limitation is that information on medications at the time of death was not available, which could have influenced receptor expression and thus cannot be excluded as a confounding factor.

An important consideration is whether the observed reduction in CB1R expression merely reflects neuronal loss in the affected regions. Since CB1 receptors are predominantly expressed on neurons, a decrease in neuronal density could theoretically account for lower CB1R levels. However, the absence of a correlation between NeuN‐positive cell counts and CB1R immunoreactivity suggests that the reduction in CB1R expression is not solely due to neuronal loss, but rather reflects a disease‐associated downregulation of receptor expression. Nonetheless, future studies should incorporate single‐cell or cell type–specific analyses to validate and further elucidate this interpretation.

Notably, CB1 receptors are widely distributed throughout the brain parenchyma [94] and are expressed not only in neurons but also in astrocytes, oligodendrocytes, and microglia [95, 96, 97, 98]. Therefore, our findings imply that disease‐related mechanisms likely contribute to the observed reduction in CB1R expression beyond general neurodegeneration.

In conclusion, we observed a significant reduction in cannabinoid CB1 receptor immunoreactivity in the hippocampus and cortex of both AD patients and AD mouse models. While no association was found between CB1R expression and cognitive performance, CB1R levels negatively correlated with both Aβ and tau pathology. These findings, based on post‐mortem human brain tissue and complementary data from mouse models, support a potential role of the CB1 receptor in the pathophysiology of AD. However, further studies are needed to elucidate the exact relationship between CB1R expression and AD‐related pathological changes. Moreover, it remains to be investigated whether CB1R expression could serve as a sufficiently sensitive marker to distinguish between mild cognitive impairment (MCI) and AD. In particular, the use of CB1‐specific PET radioligands could enable in vivo characterization of CB1R expression and help determine whether it may serve as a diagnostic marker in AD [99]. Furthermore, the question of whether AD pathology directly alters CB1R expression—or whether this relationship is mediated by indirect mechanisms—remains to be clarified. However, our findings strengthen the hypothesis that pharmacological stimulation of CB1R might offer therapeutic benefits in AD.

## Author Contributions

N.B. performed experiments, analyzed data, and wrote the manuscript. A.V.P., A.S., and H.H. performed experiments. Y.B. conceived and designed the project, performed experiments, analyzed data, and wrote the manuscript. All authors contributed to revising the manuscript and approved the final version.

## Funding

This study was supported by the “Alzheimer Stiftung Göttingen” to Y.B.

## Ethics Statement

All experiments involving human tissue were approved by the Ethics Committee of the University Medical Center Göttingen (protocol number 12/1/15). All animal procedures complied with German animal welfare regulations and were approved by the local authorities (LAVES) [15/1760, 16/2364, 14/1450].

## Conflicts of Interest

The authors declare no conflicts of interest.

## Supporting information


**Figure S1:** Representative immunofluorescence images of AD human brain tissue. (A) Representative images showing astrocytes labeled with GFAP and (B) microglia labeled with Iba1 in the superior temporal gyrus of an AD patient. (C) Representative NeuN staining used to identify neuronal nuclei. (D) Quantification of NeuN‐positive cells in the superior temporal gyrus, middle frontal gyrus (MFG), and hippocampus (HI) revealed a significant reduction in AD compared to controls. Data are presented as mean ± SEM. *p < 0.05, ***p < 0.001. HI, hippocampus; MFG, medial frontal gyrus; STG, superior temporal gyrus.

## Data Availability

The datasets used and/or analyzed during the current study are available from the corresponding author on reasonable request.
