# Supplementary material for: Reduced CB1 Cannabinoid Receptor Expression in Alzheimer's Disease and Transgenic Mouse Models
Source: Aging Med (Milton). 2026 Apr 24;9(2):168–84. doi: 10.1002/agm2.70080 (PMC13163938; doi:10.1002/agm2.70080)
Supplement: Supplementary file 1 — Figure S1: Representative immunofluorescence images of AD human brain tissue. (A) Representative images showing astrocytes labeled with GFAP and (B) microglia labeled with Iba1 in the superior temporal gyrus of an AD patient. (C) Representative NeuN staining used to identify neuronal nuclei. (D) Quantification of NeuN‐positive cells in the superior temporal gyrus, middle frontal gyrus (MFG), and hippocampus (HI) revealed a significant reduction in AD compared to controls. Data are presented as mean ± SEM. *p < 0.05, ***p < 0.001. HI, hippocampus; MFG, medial frontal gyrus; STG, superior temporal gyrus. [file AGM2-9--s001.docx]

**Supplement**


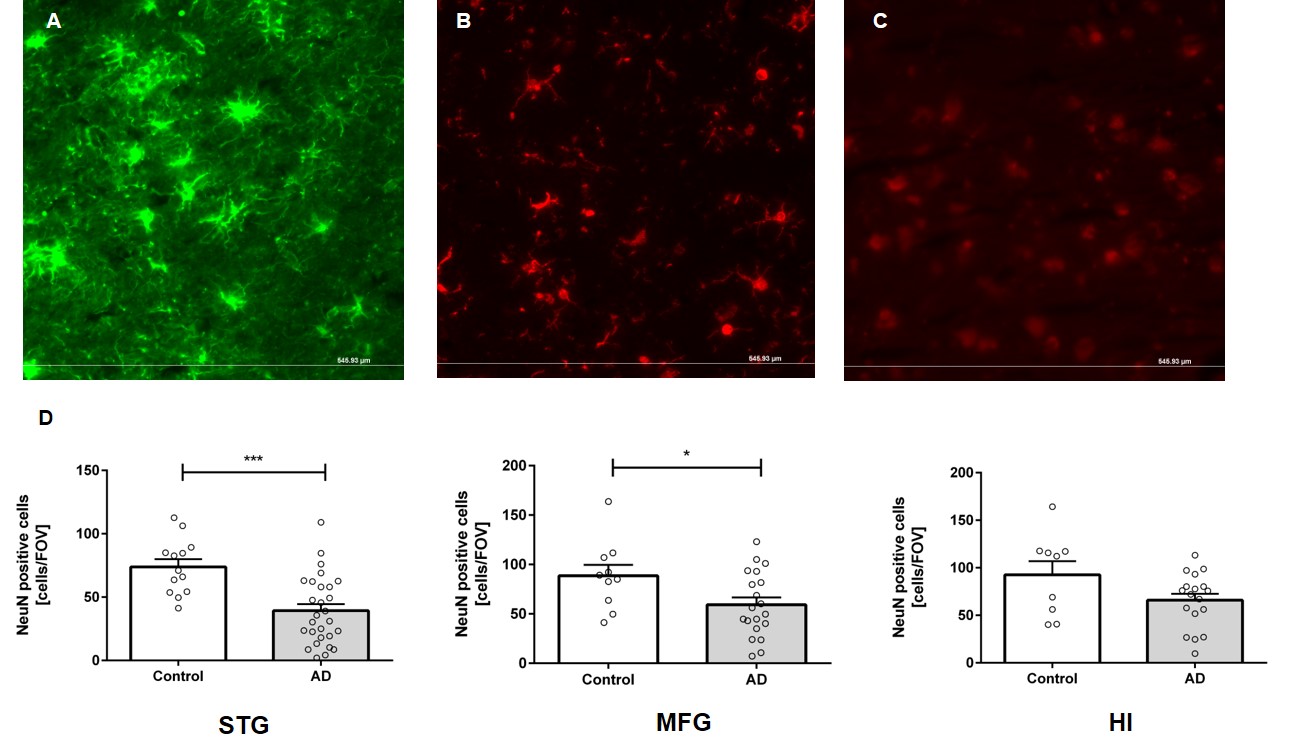


**Figure 1. Representative immunofluorescence images of AD human brain tissue.** (**A**) Representative images showing astrocytes labeled with GFAP and (**B**) microglia labeled with Iba1 in the superior temporal gyrus of an AD patient. (**C**) Representative NeuN staining used to identify neuronal nuclei. (**D**) Quantification of NeuN-positive cells in the superior temporal gyrus (, middle frontal gyrus (MFG), and hippocampus (HI) revealed a significant reduction in AD compared to controls. Data are presented as mean ± SEM. *p < 0.05, ***p < 0.001. Abbreviation: HI = hippocampus; MFG = medial frontal gyrus; STG = superior temporal gyrus.
